# Floral Scent Chemistry and Pollinators of a Sexually Dimorphic Neotropical Orchid

**DOI:** 10.3390/plants12010017

**Published:** 2022-12-20

**Authors:** Paulo Milet-Pinheiro, Carlos E. Pinto, Daniela M. A. F. Navarro, João B. F. Silva, Katharina Brandt, Manfred Ayasse

**Affiliations:** 1 Laboratório de Interações Ecológicas e Semioquímicos, Universidade de Pernambuco, Campus Petrolina, Rodovia BR 203, Km 2, Petrolina 56328-903, Brazil; 2 Laboratório de Ecologia Química, Departmento de Química Fundamental, Universidade Federal de Pernambuco, 1235 Avenida Professor Moraes Rego, Recife 50670-901, Brazil; 3 Collaborator of the Departamento de Botânica, Goeldi Museum, Av. Magalhães Barata 376, Belém 66040-170, Brazil; 4 Institute of Evolutionary Ecology and Conservation Genomics, University of Ulm, 89081 Ulm, Germany

**Keywords:** *Catasetum*, orchid bees, Orchidaceae, perfume flowers, pollination

## Abstract

*Catasetum* is a speciose Neotropical orchid genus of which male and female flowers emit scents acting both as attractant and reward for their exclusive pollinators, male orchid bees (Euglossini: Apidae). In *Catasetum*, it is well known that flowers display a remarkably morphological sexual dimorphism. However, it remains poorly investigated whether this is also true for floral scents. Here, we investigated the pollination ecology and floral scent traits (chemistry and total emission) of *C. maranhense*, a species endemic to the Brazilian N/NE region. Males of *Euglossa securigera* are the only pollinators of *C. maranhense*. The floral scent of *C. maranhense* is composed of 29 volatile compounds, with eucalyptol, indole, (*E*)-Methyl p-methoxycinnamate, and (*Z*)-Methyl p-methoxycinnamate accounting for more than 80% of the scent bouquet. No sexual dimorphism was detected in any of the traits investigated. We discuss the ecological and evolutionary significance of our findings to *Catasetum* species and other unisexual perfume plants.

## 1. Introduction

In Neotropical forests, flowers of around 1000 plant species of no less than 15 families [1] are very unusual in terms of the reward they offer to pollinators. Instead of nectar or pollen, they produce floral scents that act as both an attractant and a reward for pollinators (the “perfume flower” syndrome as proposed by [2]). The floral scents are collected exclusively by males of the bee tribe Euglossini [3], which comprises about 250 species belonging to five genera [4]. Euglossine males developed morphological adaptations to collect and store the floral scents [3,5], which are used during courtship display in perching sites, probably to convey information about their quality (fitness) and/or identity (species) to females [6,7].

*Catasetum* Rich. ex Kunth. (Orchidaceae) is the most diverse genus pollinated by male euglossine bees, including ca. 170 species [8] that are distributed from southern Mexico to northern Argentina [9]. It is one of the best studied Neotropical orchid genera with respect to floral scent chemistry and pollination ecology. Nevertheless, the pollinators as well as the floral scent chemistry of >75% of all species have never been investigated [10]_._ Out of the species for which good information is available, bees of the genera *Euglossa* and *Eulaema* are the most frequent pollinators, with only a few reports of species pollinated by *Eufriesea*. When considering the floral scent chemistry, about 20 species had their scent bouquet satisfactorily identified (i.e., >90% of the total discharge). From these works, it is clear that the scent bouquet of each species is usually dominated by two to three major compounds that are commonly reported across species, particularly 1,8-cineole, α-pinene, (E)-dihydrocarvone, (E)-carvone epoxide, carvone, and ipsdienol [10].

Differently from most orchids, *Catasetum* species have unisexual flowers presenting a pronounced morphological dimorphism that is directly involved in a specialised pollination system [11]. Male flowers generally have a catapult-like apparatus that releases the pollinarium after a male euglossine bee collects the scents at the labellum. Then, the pollinarium is attached to the pollinators body, and the bee is forcibly expelled from the flower and flies away (for a schematic drawing, see [12]). The forcible pollinarium emplacement has been described as a traumatic event for the pollinators [13]. According to these authors this traumatic event could promote the morphological dimorphism of flowers because euglossine males might avoid male flowers after being shocked.

More recently, this event has been proposed to also promote sexual dimorphism in floral scents [10]. This idea is founded on the capacity of male euglossine bees to change their scent-foraging motivation after collecting a given compound intensively, probably to keep a species-specific signature in their perfume blends [14]. In this case, divergence in floral scent traits (e.g., chemical composition and amount of scent emission) may be adaptive if this induces scent-seeking male euglossine bees to alternately visit female and male flowers, and if this results in increased outcrossing and reproductive success [10]. The sexual dimorphism of floral scents is well known and plays a key role in many pollination systems [15,16]. However, in perfume flowers, so far only two studies investigated this, and these studies revealed contrasting results, either presenting a sexual dimorphism (*Catasetum arietinum* [17]) or not (*Catasetum uncatum* [18]). Clearly, these findings underline how important it is to investigate a potential sexual dimorphism in floral scents in further species of the genus *Catasetum*.

**Figure 1:**
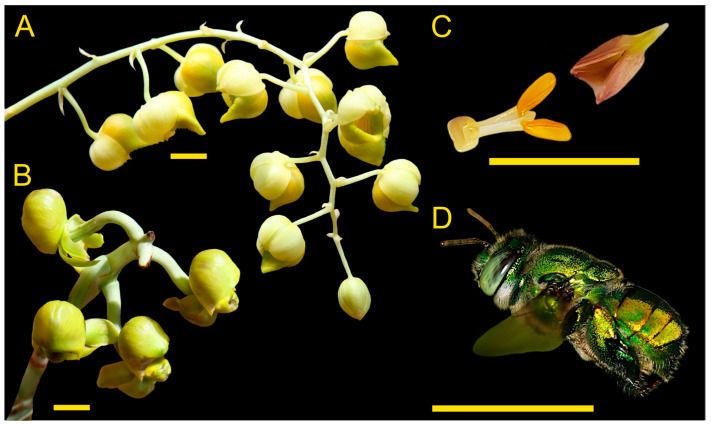
Overview of the *C. maranhense* pollination system: (**A**) male inflorescence, (**B**) female inflorescence, (**C**) pollinarium with two pollinia, and (**D**) male *E. securigera* bee. Yellow scale bars: 1 cm. Photos (**A**,**B**) by JBFS, (**C**) by KB, and (**D**) by PMP.

*Catasetum maranhense* is an epiphytic species, endemic to N/NE-Brazil, occurring in the states of Pará, Maranhão, and Piauí, i.e., in areas of the Amazon, as well as the Cerrado, and Caatinga domains [19]. Across the area of its distribution, it grows exclusively on “Babaçu” palms (*Attalea speciosa*, Arecaceae). Individuals of *C. maranhense* produce unisexual inflorescences with male and female flowers showing a remarkably morphological dimorphism (Figure 1A,B). Although quite common, information about both pollinators and floral scent traits of this species is still missing [10]. In this study, we investigated the floral scent chemistry of *C. maranhense*, as well as its interaction with male euglossine bees. We addressed two main questions: (1) Which are the pollinators? (2) Is there a sexual dimorphism in floral scent in terms of total amount and chemical composition?

## 2. Results and Discussion

### 2.1. Observation of Floral Visitors

During our focal observations, we recorded a total of thirty-seven green bees of the genus *Euglossa* on inflorescences of *C. maranhense*. On the female and male inflorescences, we recorded a total of nine and twenty-eight bees, respectively. Two of the bees visiting female flowers had a pollinarium (Figure 1C) attached to their thorax. Of the thirty-seven individuals observed, nine were collected (five on male and four on female flowers) and identified as *Euglossa securigera* (Figure 1D).

The individuals of *E. securigera* displayed a similar perfume-collecting behaviour in both male and female flowers. The bees hovered in front of the flowers, landed at the column or labellum, and then scratched the inner surface of the labellum. At this moment, in male flowers the bees eventually contacted the antennae, triggering the ejection of the pollinarium (the catapult-like mechanism) which adhered to their thorax through the sticky viscidium. In female flowers, after collecting the scent in the labellum, the bee backed out the flower and, during this movement, the pollinia could be inserted into the stigmatic cleft. Based on their behaviour, males of *E. securigera* show characteristics, which indicate they can be classified as effective pollinators of *C. maranhense*. First, they were frequent and unique visitors of *C. maranhense* flowers and second, they visited both male and female flowers, removing and depositing the pollinia.

### 2.2. Characterisation of the Floral Scent Traits of Catasetum maranhense

*C. maranhense* flowers produce a strong and sweet perfume, easily detected by the untrained human nose at a distance of about 1 m. The scent bouquet of *C. maranhense* is composed of twenty-nine compounds, belonging to four compound classes: aromatics (six compounds), monoterpenes (thirteen), N-bearing compounds (one), and sesquiterpenes (four). Five compounds could not be identified, nor attributed to any class. Monoterpenes were the dominant compound class in male and female flowers of *C. maranhense*. Eucalyptol was the main compound, followed by indole, and (*E*)- and (*Z*)-Methyl p-methoy-cinnamate. Together, these compounds accounted for more than 80% of the scent bouquet of *C. maranhense* (Table 1; see Table S1 for a complete list of compounds in individual samples).

These dominant compounds are very common among perfume flowers [20,21,22], especially in *Catasetum* species [10,17,18,23]. Eucalyptol is a common constituent of scents of perfume flowers, and is also the dominant compound in the floral scent of *C. uncatum* [18]. This compound is known as one of the most potent attractants of male euglossine bees, including *Euglossa securigera* [24,25]. Among the other major compounds, (*Z*)-Methyl p-methoxycinnamate was also dominant in floral scents of male flowers of *C. arietinum* (~20%) [17], a species which is also pollinated by *E. securigera*. On the other hand, (*E*)-Methyl p-methoxycinnamate and indole contributed in low ratios to other floral scents of *Catasetum* [17,23]. Here, indole is reported as a major component of *Catasetum* for the first time. Apart from eucalyptol, the functionality of other major compounds emitted by flowers of *C. maranhense* in attracting *E. securigera* remains to be tested. However, since these compounds were reported as potent attractants of other male euglossine species [24], it is very likely that they are also attractive to *E. securigera*.

**Figure 2:**
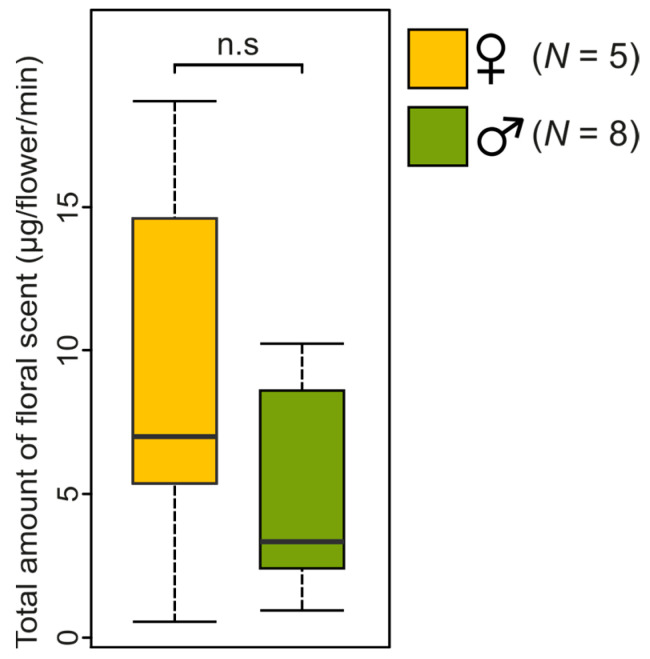
Comparison of the total amount of floral scent emitted by male and female single flowers of *C. maranhense* (Median, 25%–75%, confidence interval of 95%). n.s. indicates no significant difference at *p* > 0.05 (PERMANOVA).

**Figure 3:**
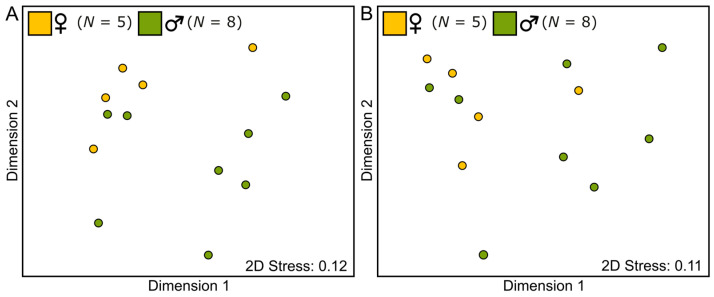
“Scent space” representation of male and female flowers of *Catasetum maranhense* visualised in two dimensions using non-metric multidimensional scaling (NMDS). (**A**) Semi-quantitative comparison based on Bray–Curtis similarity matrix (PERMANOVA: Pseudo-F_1,11_ = 1.91, *p* = 0.09; fourth root transformation). (**B**) Qualitative comparison based on Sørensen resemblance matrix (PERMANOVA: Pseudo-F_1,11_ = 1.88, *p* = 0.13; presence/absence transformation). Symbols represent the average scent pattern for each individual.

Statistical comparisons revealed that the total amount of scent emitted by female and male flowers was similar (Wilcoxon rank sum test: *W* = 26, *p*-value = 0.42; Figure 2). When considering individual components, we observed that only three of the twenty-nine compounds making up the floral scent bouquet of *C. maranhense* differed significantly, with female flowers emitting more α-thujene (Z = 2.07, *p*-value = 0.04), β-pinene (Z = 2.25, *p*-value = 0.02) and γ-terpinene (Z = 2.59, *p*-value = 0.007) than male ones (see Table S1 for the statistical outputs of all individual compounds). At last, but not least, the multivariate analyses did not reveal significant difference in the floral scent composition between male and female flowers, neither in the relative proportions of individual compounds (semi-quantitative PERMANOVA: Pseudo-F_1,11_ = 1.91, *p*-value = 0.09; Figure 3A) nor in the presence/absence of compounds (qualitative PERMANOVA: Pseudo-F_1,11_ = 1.88, *p*-value = 0.13; Figure 3B). In a general manner, the statistical analyses point to no sexual dimorphism in floral scents of *C. maranhense*. Although a difference for three individual compounds was detected, it is very unlikely that these are used by bees to discriminate flowers of different sexes, because they were emitted in very low amounts and were not ubiquitous in all samples of female and male flowers.

Similar to our findings, Milet-Pinheiro et al. [18] reported no sexual dimorphism in any of the investigated floral scent traits of *C. uncatum*. In contrast, the study by Brandt et al. [17] revealed a clear sexual dimorphism in the floral scent composition of *C. arietinum*. These contrasting results raise some interesting interpretations, mainly when considering that sexual dimorphism in flower morphology (and usually in colour) is a rule in *Catasetum* [13]. This suggests that the selective pressure fostering sexual dimorphism in morphology of *Catasetum* flowers in general does not necessarily act in the same way on floral scents. According to Romero and Nelson [13], after being shocked by the forcible pollinarium emplacement, euglossine pollinators avoid male but not female flowers, a behaviour that increases reproductive success and could therefore account for the ubiquitous morphological sexual dimorphism in *Catasetum*. While this same evolutionary mechanism could explain the sexual dimorphism of floral scents of *C. arietinum* [17], the absence of sexual dimorphism in scents of both *C. maranhense* (this study) and *C. uncatum* [18] suggest that other selective pressures might come into play to keep scent of female and male flowers similar. The attraction of specific male euglossine bees is known to depend strongly on the specific scent composition of perfume flowers [26,27]. From this perspective, the selection for dimorphism in floral scents is only possible if changes in scent composition do not result in flowers of different sexes attracting different euglossine species, which would result in reproductive failure. Consequently, pollination systems of unisexual flowers could ensure pollen transfer for the host plant through the similarity of the floral scents, which should guarantee a floral constancy of the pollinators [15,28]. Thus, the occurrence of sexually mono- and dimorphic scent patterns within the genus *Catasetum* might reflect the distinct selective pressures as a consequence of olfactory/behavioural biases of specific euglossine pollinators [10,17,29]. 

## 3. Materials and Methods

### 3.1. Observation of Floral Visitors

Floral visitors of *C. maranhense* were observed in situ at the municipalities of Esperantinópolis, Presidente Dutra and Santo Antônio de Lopes in the state of Maranhão, and in Terra Santa in the state of Pará. For five non-consecutive days, we conducted observations on three male and two female inflorescences, totalling 30 h of observation. During the observations, we described the behaviour of floral visitors on flowers and recorded if they removed/deposited the pollinaria. Some of the floral flower-visiting insects were collected using an entomological net and placed in glass pots containing ethyl acetate. The insects were then mounted with entomological pins, labelled, and identified by a specialist (J. E. Santos Júnior) using taxonomic keys [4,30]. Specimens were deposited in the entomological collection of UPE. 

### 3.2. Sampling of Floral Scents

Floral scent samples of *C. maranhense* were collected using dynamic headspace methods for two purposes: (1) to chemically characterise its scent bouquet and (2) to compare scent traits (i.e., chemical composition and total amount) in female and male flowers. Single male (n = 8) and female (n = 5) flowers, each from a different individual were individually enclosed with polyester bags (Toppits^®^, Minden, Germany), and the scented air was drawn for 3 min through an adsorbent filter (ChromatoProbe quartz micro vials; 15 mm × 2 mm i.d.; containing 1.5 mg Tenax-TA (mesh 60–80, Supelco, Bellefonte, PA, USA) and 1.5 mg Carbotrap (mesh 20–40, Supelco, Bellefonte, PA, USA); fixed using glass wool). The adsorbent filter was connected to a membrane pump (G12/01 EB, Rietschle Thomas, Puchheim, Germany) using silicone tubing. The pump worked at a constant flow rate of 200 mL/min. All samples were collected around 09:00 a.m., which was the time of highest scent emission as perceived by the human nose. The number of samples per sex depended on the availability of flowering individuals. To detect environmental contaminants, negative controls (empty bags) were collected simultaneously at a distance of ca. 2 m from the target inflorescence using the same methods described above. The samples were kept in the freezer at −20 °C until the chemical analyses.

### 3.3. Chemical Analysis

The floral scent samples of *C. maranhense* were examined on a gas chromatograph coupled to a mass spectrometer (GC/MS; Agilent 7890A™ gas chromatography, Agilent 5975C Series MSD™ mass spectrometer; Agilent Technologies, Santa Clara, CA, USA, Palo Alto, Santa Clara, CA, USA), equipped with a non-polar HP-5 ms column (Agilent J&W; 30 m × 0.25 mm i.d., 0.25 μm film thickness) and a thermal separation probe (TSP, Agilent Technologies). For details on the GCMS configuration see [23]. 

The floral scent compounds of *C. maranhense* were identified via comparing the mass spectra and retention indices with those of authentic standards accessible from commercial mass spectral libraries (MassFinder 4, NIST08, Wiley Registry™ v. 9) and ADAMS (2007) integrated in the software Agilent MSD Productivity ChemStation (Agilent Technologies, Palo Alto, USA). If possible, the identity of the compounds was checked by means of retention times and mass spectra of authentic standards. The peak areas on the chromatograms were integrated to obtain the total ion signal and their values were used to determine the relative amount (percentages) of each compound in the floral bouquet.

The absolute amount of floral compounds in the samples was quantified via injecting 100 ng of external standards belonging to different compound classes (aromatics: methyl salicylate (≥99%; Merck, Darmstadt, Germany); monoterpenes: eucalyptol (99%; Merck); and sesquiterpenes: (*E*)-β-caryophyllene (≥98%; Dragoco, Holzminden, Germany) for thermal desorption and analysing the standards in the same way as the flower scent samples. After five runs an average peak area (per compound) was calculated and used to determine the total amount of each compound in the floral samples. Volatiles detected in the control samples acquired from the environment were considered ambient contaminants.

### 3.4. Statistical Analysis

Total amount in scent emission: In order to check whether flowers of different sexes emitted different amounts of scent, we performed a Wilcoxon rank sum test with continuity correction in the software R using the standard R package “stats” v. 4.2.0 (Vienna, Austria), after checking for normality distribution of data. In addition to the comparison of the total emission, we compared the amounts in which each single compound was emitted by male and female flowers of *C. maranhense*. Depending on the normality distribution of data (Shapiro–Wilk normality test) we performed either a two sample *t*-test or an exact Wilcoxon–Mann–Whitney test [31].

Chemical composition of floral scent: We also compared whether the chemical composition of male and female flowers differed (PERMANOVA one-level design; factor: sex; 9999 permutations). For this, we followed the same statistical procedure as described in [17] to compare the scent of male and female flowers in semi-quantitative (relative amounts of compounds; Bray–Curtis’ similarity indices based on fourth-root transformed data) and qualitative (presence/absence of compounds; Sørensen’s resemblance matrix based on presence/absence transformed data) multivariate permutational analyses. All permutational analyses were run in the software PRIMER 6 (version 6.1.15; PRIMER-E Ltd., Plymouth, United Kingdom, 2012) in combination with the add-on PERMANOVA + (version 1.0.5; PRIMER-E Ltd., Auckland, New Zealand, 2012).

## 4. Conclusions

The few studies on sexual dimorphism of floral scents of *Catasetum* species performed so far show contrasting results. As in other plants with unisexual flowers, in perfume plants there are arguments for the occurrence of sexual dimorphism of scents, or for the lack of it. Here we detected no differences in the floral scents of male and female flowers and concluded that this similarity might contribute to ensure that pollinators frequently fly among conspecific flowers. On the other hand, sexual dimorphism in the floral scents of *Catasetum* species could promote flights of male euglossine bees between flowers of different sexes, because these bees become satiated when they intensively collect a given compound and look for a diversity of compounds to build their species-specific perfume mixture. In this way, the sexual dimorphism of floral scents could promote flights between male and female flowers. Although in recent years considerable advances have been made to understand the patterns of scent in unisexual *Catasetum* flowers, a broader study including not only further *Catasetum* species but also other perfume plants with unisexual flowers (e.g., *Cycnoches* and *Clowesia*), will greatly help us to understand the evolution of mono- or dimorphic scents in unisexual perfume plants.

## Figures and Tables

**Table 1:** Relative contribution of volatile compounds in headspace samples of male (n = 8) and female (n = 5) flowers of *C. maranhense*. Volatiles are grouped in compound classes and listed according to elution on an HP-5 column. RI: retention index. * Identification based on authentic standards. Chemical compounds with mean of emission rate < 0.01% and > 0% are considered a trace amount and identified as Tr.

Compounds	RI	Male (% Mean ± SD)	Female (Mean ± SD)
**Aromatics**			
1,4-Dimethoxybenzene *	1168	0 ± 0	Tr
Methyl p-anisate *	1376	2.28 ± 2.46	0.69 ± 0.56
(E)-Methyl cinnamate *	1386	0.80 ± 0.97	1.23 ± 0.59
Ar-curcumene	1486	0.17 ± 0.20	0.27 ± 0.36
(Z)-Methyl p-methoxycinnamate	1592	6.07 ± 4.63	14.46 ± 9.54
(E)-Methyl p-methoxycinnamate	1675	10.38 ± 11.10	18.66 ± 23.97
**Monoterpenes**			
α-Thujene *	927	Tr	0.07 ± 0.06
α-Pinene *	932	1.46 ± 0.96	1.56 ± 1.38
Camphene *	947	Tr	Tr
Sabinene *	972	0.51 ± 0.73	0.90 ± 0.68
β-Pinene *	974	0.03 ± 0.10	0.25 ± 0.17
β-Myrcene *	992	3.92 ± 2.13	5.52 ± 3.34
Limonene *	1028	1.96 ± 2.88	0.97 ± 0.69
Eucalyptol *	1030	53.27 ± 20.71	36.64 ± 21.70
(Z)-β-Ocimene *	1040	Tr	0.02 ± 0.04
(E)-β-Ocimene *	1050	0.96 ± 0.94	1.11 ± 1.03
γ-Terpinene *	1058	Tr	0.08 ± 0.05
α-Terpinolene *	1088	0.02 ± 0.04	0.01 ± 0.02
α-Terpineol *	1191	0.15 ± 0.24	0.07 ± 0.17
**N-bearing compounds**			
Indole *	1293	13.75 ± 11.10	13.57 ± 7.08
**Sesquiterpenes**			
Dihydro-β-ionone *	1439	0.10 ± 0.03	0.05 ± 0.09
(Z,Z)-α-Farnesene	1494	0.04 ± 0.07	0.04 ± 0.02
(E,E)-α-Farnesene	1507	3.29 ± 2.42	3.40 ± 2.57
Sesquicineole	1516	0 ± 0	0.06 ± 0.10
5 unknown compounds		0.81 ±1.51	0.24 ± 0.36

## Data Availability

The data presented in this study are available within the article or Supplementary Material.

## Supplementary Materials

The following supporting information can be downloaded at: https://www.mdpi.com/article/10.3390/plants12010017/s1, Table S1: Relative contribution of volatile compounds in headspace samples of male (n = 8) and female (n = 5) flowers of *C. maranhense*.
